# Facts and Conjectures on the Trembles and Milk-sickness

**Published:** 1842-09

**Authors:** 


Art. II.—*Facts and Conjectures on the Trembles and Milk-sickness.* In a letter from Dr. G. B. Taylor of Morganfield, Kentucky, to Dr. W. L. Sutton, of Georgetown in this State; communicated by the latter with some remarks.

* * * The subject of your letter is one on which I have made many inquiries, and in every neighborhood within

---

my reach; especially since the death of Wm. Hopgood, with whom you were well acquainted: he died at Highland about three years since. As he was a young man of close observation, and possessed more than common discrimination, I was disposed to place some confidence in his opinion of the producing cause of his disease; and I never saw a man more confident of any thing being the cause of his sickness, than he was that his was produced by mineral water, which he drank in the Highland hills, and at the very place where most of our cattle and horses have died. I did not see Hopgood until three or four days after his attack. When I visited him first, he was salivated, and his bowels had been freely acted on by oil, &c. He complained of feeling very tired, and although naturally neat, would not turn his head to spit, so as to avoid spitting on his bed. The action of the heart was so strong, that you could see the whole system shake at every pulsation. The pulse was slow and strong, not more than fifty strokes to the minute after I saw him; and I could count the strokes by putting my hand on the top of his head. I bled him freely three times, and he grew worse after each bleeding. He was disposed to faint when bled, and upon recovering from syncope, would vomit, and in a short time after, the pulse would be as strong as ever. I think he died upon the second day after I saw him. * * * * * I have been in the habit of making inquiry of intelligent persons in the different neighborhoods, where they are liable to the disease; and I find the same mineral exists in every neighborhood—one in Union, two in Henderson, and three in Daviess counties. In all those places there are sweet springs, the water running from thin strata of stone coal, which contains so much sulphate of iron, that the smiths cannot use it without overhauling and throwing out the mineral. All those places are in the immediate neighborhood of sugar-tree hills, and I think an unusual growth of poison oak. I am satisfied myself that the disease is produced by the mineral, and that it contains a portion of arsenic. There is also some petroleum on the surface of the water at these sweet springs; and stock resort to them as *licks*, for want of salt. * * * * *. There is no country I have visited, that contained more poison oak, than that rich section in which you are situated, until it was destroyed by the stock. Horses and cattle feed on it with avidity, wherever it grows, and with impunity. I have just settled in the woods where the poison oak is very abundant. I have no stable as yet, and my riding horse has skinned every poison oak vine I can see, as high as he can reach. There have been several trees cut down with large vines on them, and he has peeled them the whole length, where he could get at them, and devoured all the small branches. I believe that females are not so liable to this disease during lactation, though not entirely exempt; as there was an instance at Leonard's, when he lived on the hill at Highland, of a cow and calf dying about the same time—the cow running in a stalk field, and the calf in a calf lot. There are two distinct vines called poison oak—the five-leaved and three-leaved. Both climb trees, and very much resemble each other. The poison kind has three leaves on the stem and puts out more radicles, attaches itself more firmly to the tree, and bears a small bunch of berries, very much resembling grapes, but smaller and blue. I have known of children eating them twice since I can recollect. It did not prove fatal in either of the cases; but both were very distressing, and neither of the subjects could see for a week or more. And they were certainly affected very differently from the cases of milk-sickness which have come under my observation. The poison oak will not poison all persons, which any farmer will tell you. Some can cut a green vine, and mark their shirts while on them, with impunity. I was confined near a month, when a boy, by doing so, and was as full of blisters all over my body, as ever any person was of pustules from small-pox. I have conversed with several persons who have had the milk-sickness, some at different times. They all say they feel tired constantly, and are incapable of taking much exercise, until they have been freely purged, which generally cures slight attacks. They consider calomel injurious. Those who have been hard at work about the time of attack, rarely recover.

Senna, salts and jalap are considered among the best remedies. I think some of the preparations of iron would be good. Buzzards will not feed on the carcass unless compelled by hunger. I have noticed them sit upon trees around and watch the carcass until it rotted, without breaking the hide. But I have seen them, when they had been pressed by hunger, lying dead around the carcass. Some slowly expanded their wings, on my approach, as if about to fly, but, at the effort to rise, would tumble forward, unable to raise themselves. You know that we, at one time, had a fine pack of dogs. We frequently lost them by their eating carrion in the neighborhood of Highland. Some would come home with the appearance of being foundered, but were never fit for the chase afterwards, as they would take the 'tires' as it is generally termed; and but few horses or oxen are of much use, after having the disease badly. Mr. A. Silver once lived at Highland, and said he could cure his cattle, if he saw them in time, by giving them enough of the decoction of white walnut to purge them. Peter Rives, at one time, was under the impression that arsenic would cure the distemper in his dogs, and was in the habit of giving it freely in that disease. Several were cured of distemper, but they were of no use in the chase, and appeared to be affected precisely in the same manner as those which had the 'tires' from eating cattle that had died from milk-sickness, which is my strongest reason for suspecting arsenic in the mineral. The disease produced by poison oak (so far as has come under my observation) differs so widely from milk-sickness, that I cannot attribute the disease to that vegetable. * * * * *

Fall before last, we suffered very much for stock water, and several persons lost all the cattle they had. They died about these mineral springs in the hills at Highland, and the owners think they did not go into the bottoms. The people in the neighborhood of the disease, attribute it to something that grows in the rich sugar-tree hills exclusively; but we have the same kind of land and growth where there is no disease of the kind. I had forgotten to say that work oxen, regularly fed, are liable to the disease, at the mouth of Highland. If the attack is slight, they will recover entirely by rest—if severe, they are worthless for the yoke afterwards. If the attack is slight, very hard labor will destroy the animal. Hogs and deer are not liable to the disease that I have ever heard of.

### ADDITIONS BY DR. SUTTON.

In addition to the above, I will mention that since I commenced this letter, I have conversed with a very respectable lawyer, who has resided at Mill's Point, Ky., and at a place in Tennessee, where also the milk-sickness was common. He says that in both neighborhoods, the people ascribed the origin of the complaint to the water; that the disease was much more common in dry seasons; and that, at such times, certain springs blamed with its production, acquired a taste which was not observable when the water was flush. He gave a number of facts, tending to confirm the general opinion, but which would swell this communication to an unwarrantable length. Upon the whole, they corresponded mainly with those held by Dr. Taylor. Again, I was informed by the widow of a Dr. Rainey, who resided somewhere not far from Covington, and who had a good deal of practice in the disease, that he had under his care a man and his wife (perhaps both died, but of this I am not positive), who had abstained entirely from milk and its products for a long time, for fear of the disease, in whom, nevertheless, it was well marked. Of course I can say nothing as to the accuracy of his observations, or the faithfulness of the report. The lady is quite intelligent, but there are a thousand notions upon the subject; and I cannot say that I am satisfied there is any such specific disease.

*February*, 1841.

